# Topical Administration of a Nanoformulation of Chitosan-Hyaluronic Acid-Epoetin Beta in a Rat Model of Glaucoma

**DOI:** 10.3390/ph16020164

**Published:** 2023-01-23

**Authors:** Beatriz Silva, Lídia M. Gonçalves, Berta São Braz, Esmeralda Delgado

**Affiliations:** 1 CIISA—Centre for Interdisciplinary Research in Animal Health, Faculty of Veterinary Medicine, Universidade de Lisboa Avenida da Universidade Técnica, 1300-477 Lisbon, Portugal; 2 Associate Laboratory for Animal and Veterinary Sciences (AL4AnimalS), 1300-477 Lisbon, Portugal; 3 Research Institute for Medicines (iMed.ULisboa), Faculty of Pharmacy, Universidade de Lisboa, 1649-003 Lisbon, Portugal

**Keywords:** glaucoma, neuroprotection, nanoparticles, epoetin beta, chitosan, hyaluronic acid, ocular delivery

## Abstract

The present work investigates the effects of chitosan-hyaluronic acid-epoetin beta (CS/HA-EPOβ) nanoparticles after topical ocular administration in a rat glaucoma model. Wistar Hannover rats (*n* = 24) were submitted to a complete ophthalmological examination and electroretinography, followed by glaucoma induction in their right eye on day 1 of the study. Treatment group (T) received CS/HA-EPOβ nanocarriers (*n* = 12), while the control group (C) received only empty ones. Electroretinography was repeated on day 3 (*n* = 24) and before euthanasia on day 7 (*n* = 8), 14 (*n* = 8), and 21 (*n* = 8), followed by bilateral enucleation and histological assessment. The animals showed good tolerance to the nanoformulation. Maximum IOP values on the right eye occurred shortly after glaucoma induction (T = 62.6 ± 8.3 mmHg; C = 63.6 ± 7.9 mmHg). Animals from the treated group presented a tendency for faster recovery of retinal electrical activity (*p* > 0.05). EPOβ was detected on the retina of all treated eyes using immunofluorescence. Control animals presented with thinner retinas compared to the treated ones (*p* < 0.05). Therefore, topical ocular administration of CS/HA-EPOβ nanoparticles enabled EPOβ delivery to the retina of glaucomatous rats and promoted an earlier retinal recovery, confirming EPOβ’s neuroprotective effects. The encouraging results of this preclinical study pave the way for new strategies for topical ocular administration of neuroprotective compounds.

## 1. Introduction

Neurodegenerative ocular diseases, such as glaucoma, have a substantial impact in people’s daily lives, as they can cause major visual impairment and ultimately blindness. Glaucoma is a group of ocular disorders with an unclear pathophysiology that is accompanied by degeneration and death of retinal ganglion cells (RGC), which leads to optic neuropathy [1]. The principal risk factor is high intraocular pressure (IOP), and current glaucoma therapy is focused on managing it by increasing aqueous humor outflow and decreasing its production, in an attempt to delay the disease’s progression [2]. However, research in ophthalmology is shifting towards neuroprotection as a complementary approach to glaucoma treatment, and several neuroprotective strategies are under study, such as the use of progesterone, neurotrophic factors [3], asiatic acid [4], and gene therapy [5]. 

Erythropoietin (EPO) is a well-known glycoprotein that, apart from its hematopoietic role, can protect the retina by attenuating inflammatory responses, stimulating neurotrophin expression and up-regulating neurotrophic factors, amongst other features [6]. The antiapoptotic effects of EPO in the RGC include the down-regulation of caspase-3 through the PI3K/AKT pathway, the up-regulation of Bcl-xl [7], the suppression of the cytochrome C mitochondrial release, and intracellular calcium regulation [8]. Epoetin beta (EPOβ), a recombinant variant of EPO, has already been tested in glaucomatous rats using the subconjunctival route after its administration in an aqueous solution, revealing positive effects in the retina through a faster recovery of retinal electric responses and a decrease in cellular damage of the retina [9]. 

Following this line of research, our team developed a nanoparticulate system with chitosan and hyaluronic acid (CS/HA) to act as an ocular delivery system [10] and studied the *in vivo* safety of this nanoformulation in healthy Wistar Hannover rats after subconjunctival [11] and topical (data not published) administrations. CS/HA-EPOβ nanoparticles were described with a size of 289 ± 3 nm, polydispersity index (PdI) of 0.126 ± 0.085, and zeta potential (ZP) of 39 ± 1 mV, which was not significantly different from the empty nanoparticles (size 300 ± 6 nm; PdI 0.219 ± 0.043; ZP 33 ± 1 mV). The results of the in vitro release study in simulated tear fluid (37 °C, pH 7.4) showed that 60 to 70% of EPOβ was released in 15 min, and the remaining (until ≈ 90%) was sustained-released within 6 h. The drug loading capacity was 17.4 ± 0.1%, and the encapsulation efficiency was 38.4 ± 0.3%. Moreover, CS/HA-EPOβ nanoparticles were considered noncytotoxic for both ARPE-19 and HaCaT cells [10].

Both chitosan and hyaluronic acid are natural polymers with excellent mucoadhesive features, which can be enhanced through mutual association. Chitosan interacts with the ocular mucosa by ionic forces and is capable of widening cellular tight junctions [12,13,14], while hyaluronic acid’s mucoadhesive power is associated to CD44 receptors located both in the corneal epithelium and endothelium [15,16,17]. Therefore, despite the natural ocular defense mechanisms that delay drug permeation [14,18], CS/HA nanoparticles could enhance the retention time of drugs on the ocular surface, potentially increasing their penetration and intraocular bioavailability [19]. 

Our team has also demonstrated that after topical instillation of CS/HA-EPOβ nanoparticles in Wistar rats, EPOβ could permeate through the outer layers of the eye and reach the retina after 12 h, remaining detectable 21 days later (data not published). These results led us to consider the topical route of administration as an option to deliver a neuroprotective/neuroregenerative treatment targeting the retina in an experimental model of retinal disease. As far as we know, this innovative approach has never been reported in the literature before. Thus, in the present preclinical study, we intended to evaluate the effects of this newly developed CS/HA-EPOβ nanoformulation administered through a topical ocular route using a rat model of glaucoma. 

## 2. Results

### 2.1. Nanoparticles Characteristics

CS/HA nanoparticles containing 1000 IU EPOβ were not significantly different from empty nanoparticles, regarding their physicochemical characteristics (*p* > 0.05). CS/HA-EPOβ nanoparticles presented a size of 330 ± 15 nm, PdI of 0.174 ± 0.016, and ZP of +28 ± 1 mV, while empty nanoparticles presented a size of 359 ± 21 nm, PdI of 0.317 ± 0.022, and ZP of +37 ± 3 mV.

### 2.2. Ophthalmological Examinations and Behavior

Animals demonstrated a high tolerance to the nanoformulation administration, both to empty (control groups) and EPOβ-loaded (treatment groups) CS/HA nanoparticles. Their behavior was monitored throughout the study using the Rat Grimace Scale, the results being the same for all the animals included in the study (*n* = 24; orbital tightening “0”, nose flattening “0”, ear changes “0”, and whisker change “0”). Thus, signs of pain or discomfort were absent, meaning that the analgesic protocol was effective. Moreover, no animal reacted with discomfort during the topical administrations. 

Ophthalmological examinations revealed no severely abnormal ocular changes throughout the study, such as blepharospasm, epiphora, purulent ocular discharge, moderate/severe conjunctival hyperemia, corneal oedema, hyphema, or others. Neuroophthalmological and fundoscopic exams showed no alterations in any of the animals (*n* = 24). Biomicroscopic exams revealed that one rat from group T7 and one rat from group T21 exhibited superficial keratitis in the OD, and one rat from group C14 developed bilateral superficial keratitis, confirmed by fluorescein test. All rats recovered completely in 48 h, and no therapy was necessary. These cases could be justified by the exposure to keratitis during anesthesia since the blinking reflex was abolished. In addition, on the day after glaucoma induction, mild conjunctival hyperemia was present in the OD of all animals (rated 1/3), and a discrete amount of bilateral chromodacryorrhea was observed in 14/24 rats, from both the treatment and control groups. These ocular signs were expectable given the surgery itself and the postsurgical stress. 

**Figure 1:**
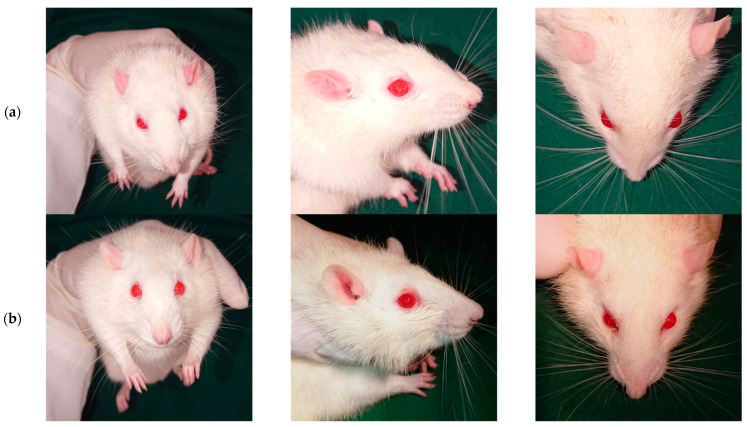
Pictures of a rat 1 h (**a**) and 3 days (**b**) after the cauterization of the 3 episcleral veins of the OD. The animal shows symmetry between both ocular globes and no signs of ocular discomfort.

Figure 1 shows pictures of a rat 1 h after the glaucoma induction (Figure 1a) and 3 days later (Figure 1b). The ocular symmetry is evident at both timepoints, showing that the IOP increase apparently had no impact in the conformation of the right ocular globe. The same was noted for the rest of the animals.

### 2.3. Intraocular Pressure

The average IOP of conscious rats, at the beginning of the study, was OD = 17.2 ± 1.4 mmHg and OS = 17.2 ± 0.9 mmHg for treatment groups and OD = 17.4 ± 1.3 mmHg and OS = 17.3 ± 1.4 mmHg for control groups, with no statistically significant differences between groups (*p* > 0.05). Those values were in accordance with the reference IOP for healthy Wistars Hannover rats [20].

Table 1 shows the IOP measurements before and after (T = 0) glaucoma induction, denoting that the difference between IOP values before and after glaucoma induction was statistically significant for all animals (*p* < 0.05). This validates this technique and means that the cauterization of three episcleral veins was an effective procedure for raising the IOP of the OD, with no indirect effects in the OS.

**Figure 2:**
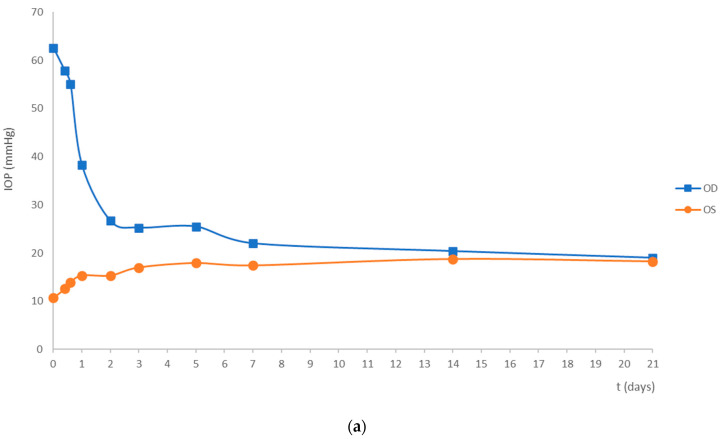

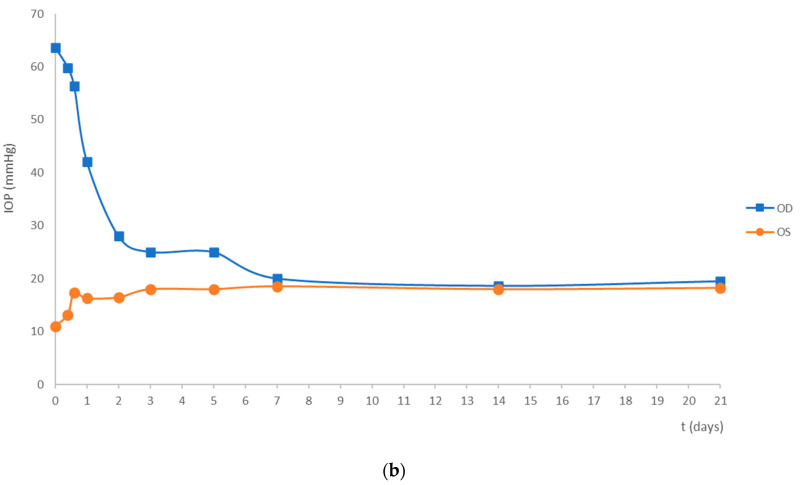
Mean IOP from the OD (orange) and from the OS (blue) since the day of glaucoma induction (t = 0) until 21 days later, in the treatment (**a**) and the control (**b**) groups. Time is presented in days in the X axis and IOP is presented in mmHg in the Y axis.

Figure 2 represents the IOP variation for the treatment (a) and the control groups (b) from the time of glaucoma induction (T0) until the end of the study (T21). The IOP of the OS varied between 10 mmHg and 22 mmHg, with an average of 15.4 ± 2.8 mmHg for treated animals and 16.1 ± 2.8 mmHg for control animals, which were within the reference range [20]. Considering the OD, the maximum IOP measured occurred at T0 (Figure 2), with statistically significant differences between OD and OS (*p* < 0.05). No difference was observed between groups in the average IOP at T0 (*p* > 0.05), being 62.6 ± 8.3 mmHg for the treatment group and 63.6 ± 7.9 mmHg for the control group (Table 1). In both groups, the IOP of the OD increased on average 3.6 folds from the physiological baseline, but rapidly decreased in the following days, reaching physiological values at day 7 (Figure 2). The treatment and control groups did not show any significant differences in the average IOP measurements (*p* > 0.05), meaning that EPOβ did not seem to influence IOP.

### 2.4. Electroretinography

Three flash electroretinographies per animal were performed to evaluate the changes in retinal function after glaucoma induction and to assess the retinal response to treatment with CS/HA-EPOβ nanoparticles. The waveforms and amplitudes of the a- and b-waves were analyzed, and the results (presented in μV as mean ± SD (min; max)) are fully described below.

#### 2.4.1. Efficacy of the Glaucoma Induction

As mentioned before, a previous ERG was performed in all animals before glaucoma induction. In order to assess the retinal damage of the OD due to the increased IOP, an ERG was performed 3 days after glaucoma induction. The analysis of the changes in retinal electrical activity sustained that the cauterization of 3 episcleral veins successfully led to retinal impairment in the OD, as statistically significant differences were observed in the majority of ERG traces, in both the treatment and control groups (*p* < 0.05) (Table 2). SLR and SA showed statistically significant differences in both the a- and b-waves in both groups, while PA, PLR, and PF presented significant differences only in the b-wave, as shown in Table 2. No statistically significant differences (*p* > 0.05) were detected between the treatment and control groups, indicating that the episcleral veins cauterization was performed similarly in all groups of animals. For instance, within the treatment group, the SLR at 5 dB presented an a-wave of 263 ± 36 (180; 312) μV before glaucoma induction and 46 ± 27 (8; 90) μV at day 3, while the b-wave was 618 ± 15 (440; 891) μV before the microsurgery and 124 ± 66 (19; 208) μV at day 3 (Table 2). Likewise, for the control group, the SLR at 5 dB presented an a-wave of 266 ± 31 (203; 300) μV before the surgical procedure and 45 ± 15 (19; 74) μV at day 3, and the b-wave was 626 ± 12 (349; 810) μV before the microsurgery and 123 ± 47 (67; 227) μV at day 3 (Table 2). Table 2 summarizes the ERG results of the treatment and control groups, before and 3 days after glaucoma induction in the OD.

**Figure 3:**
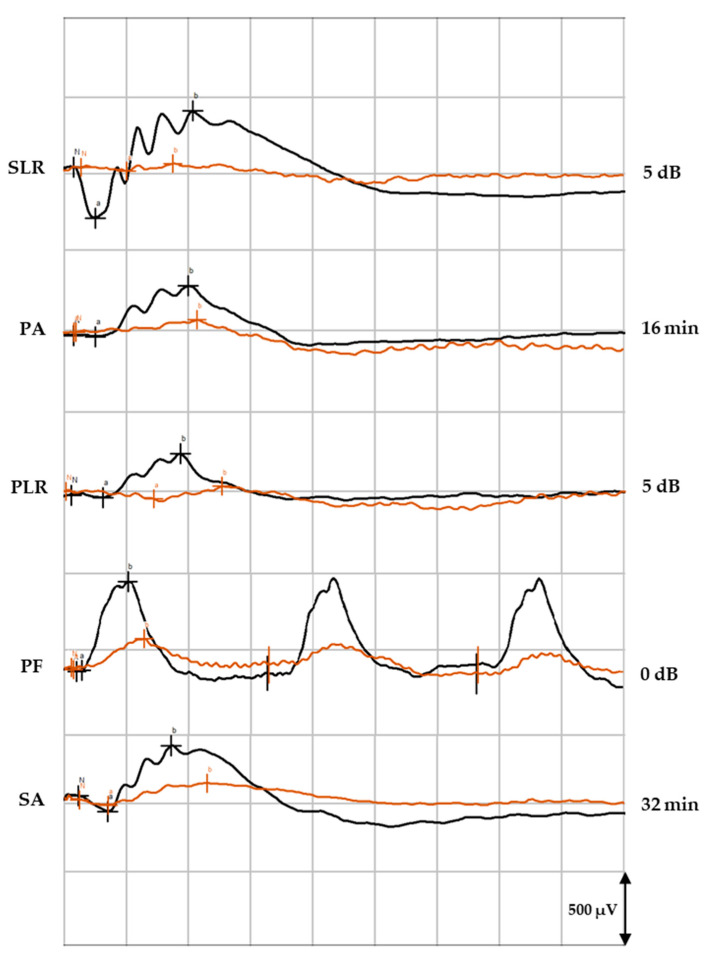
Example of ERG waveforms recorded from the OD at the beginning of the study (black) and at day 3 after the glaucoma induction (orange) in an animal from the control group. a, a-wave; b, b-wave; N, zero/basis; SLR, scotopic luminescence response; PA, photopic adaptation; PLR, photopic luminescence response; PF, photopic flicker; SA, scotopic adaptation; dB, decibel; min, minutes.

Figure 3 illustrates the waveforms recorded from the OD before the procedure and at day 3, where we can observe flattening of the waves as a result of decreased retinal electric response. On the other hand, the a- and b-waves recorded from the OS showed no statistically significant differences between day 0 and 3 days after the surgery, in neither the treatment nor the control groups (*p* > 0.05). For example, in the treated group, the SA of the OS at 32 min before the glaucoma induction had mean a- and b-waves of, respectively, 84 ± 49 (30; 212) μV and 394 ± 14 (158; 695) μV, while 3 days later, they corresponded to 87 ± 39 (31; 223) μV and 400 ± 16 (139; 795) μV, respectively. Before the procedure, the control group presented an a-wave of 90 ± 34 (39; 145) μV and a b-wave of 386 ± 85 (216; 515) μV, while 3 days later, the mean a-wave was 87 ± 53 (36; 195) μV and the b-wave corresponded to 399 ± 10 (206; 575) μV.

#### 2.4.2. Effects of CS/HA-EPOβ Nanoparticles

ERG was also used to evaluate the evolution of the retinal electrical activity after glaucoma induction, performed at timepoints 3, 7, 14, and 21 days, both in the treated and control groups, in order to assess the neuroprotective effects of CS/HA-EPOβ nanoparticles in the retina. Analyzing the results (Table 3), it is noticeable that the treated group (T) had higher b-wave amplitudes than the control group (C) from day 7 until day 21, although these differences were not statistically significant (*p* > 0.05). Differences were more evident at the end of the study (21 days), particularly in scotopic conditions. The mean b-wave of the SLR (5 dB) was 341 ± 55 μV in the treatment group and 257 ± 80 μV in the control group, while the mean b-wave of the SA (32 min) was 198 ± 51 μV in the treatment group and 145 ± 39 μV in the control group.

When studying the differences between the timepoints T3 (retinal damage baseline), T7, T14, and T21, significant differences between the treated and control groups were found. Mean values were analyzed (*n* = 4 per group/timepoint) and statistically significant differences (*p* < 0.05) were evaluated in each step of the ERG. Table 4 summarizes the comparison between groups/timepoints.

Comparing day 3 with day 7, statistically significant differences (*p* < 0.05) were detected in the treatment group in the majority of the SLR exams (from –25 dB to 5 dB), while in the control group, differences were significant only at –5 dB and 0 dB. Further, the treated group showed significant differences in almost all SA exams (from 0 to 16 min) (*p* < 0.05), while in the control group, the differences were significant at steps 0, 2, and 4 min. 

When comparing day 3 and day 14, statistically significant differences (*p* < 0.05) in the SLR were maintained between –25 dB to 5 dB in the treated group, and in the control group, the significance was observed from –10 dB to 0 dB. Both groups presented statistically significant differences at 0 dB in the PF. In the SA step, significant differences in the treated group were found during the entire procedure (0–32 min) (*p* < 0.05), and in the control group, throughout the majority of the protocol (0–16 min) (*p* < 0.05). 

Finally, when comparing day 3 with day 21, the treated group presented significant differences (*p* < 0.05) in the whole SLR step (from –35 dB to 5 dB), while in the control group, the difference was significant from –25 to –5 dB. Both groups presented significant differences (*p* < 0.05) in all SA examinations (0–32 min). Considering photopic conditions, treated groups showed statistically significant differences (*p* < 0.05) in the PA (8 and 16 min), PLR (0 and 5 dB), and PF (0, –5, –10 dB), while the control group solely maintained the significant differences in PF at 0 dB.

**Figure 4:**
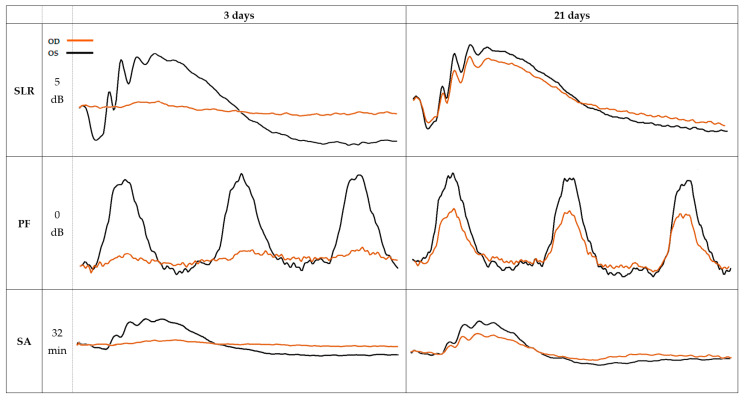
ERG waveforms of the OD (orange) and the OS (black) recorded at day 3 and day 21 after the glaucoma induction, from the treatment group. SLR, scotopic luminescence response; PF, photopic flicker; SA, scotopic adaptation; OD, right eye; OS, left eye.

Even though the overall results showed in Table 3 presented no statistically significant differences (*p* < 0.05) between the treated and the control groups, both the a- and b-wave amplitudes, with time, came closer to the basal values, with the treatment group presenting a tendency for faster retinal recovery in both the scotopic and photopic conditions (Table 4). Figure 4 illustrates some examples of b-waves from the treatment group at 3 days and at 21 days after glaucoma induction.

### 2.5. Histologic Evaluation

#### 2.5.1. Retinal Thickness

**Figure 5:**
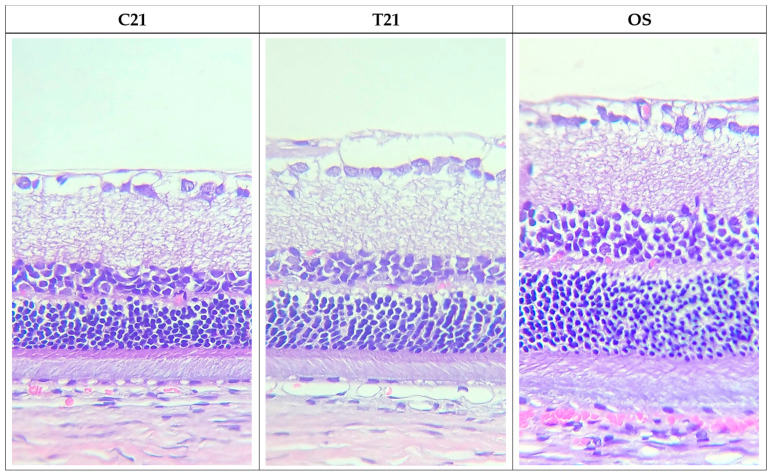
Photomicrographs of retinal sections from the OD, 21 days after glaucoma induction, from the control group (C21) and treated group (T21), and from a non-glaucomatous eye (OS; *n* = 4). We can observe that the improvement in retinal thickness was more pronounced in the treated group. Hematoxylin and eosin staining, 40× magnification.

Measurements of retinal thickness were performed in OD cross sections stained with HE, and the results are presented in Table 5 and in Figure 5. There was a gradual improvement in retinal thickness in the treatment and control groups from day 7 to day 21, which might indicate a process of retinal regeneration in both groups. Yet, animals from the treatment group presented a more pronounced improvement in retinal thickness when compared to the control group, these differences being statistically significant (*p* < 0.05). Overall, histological evaluation yielded a more expressive retinal recovery in the treated group versus the control group.

#### 2.5.2. Immunofluorescence

**Figure 6:**
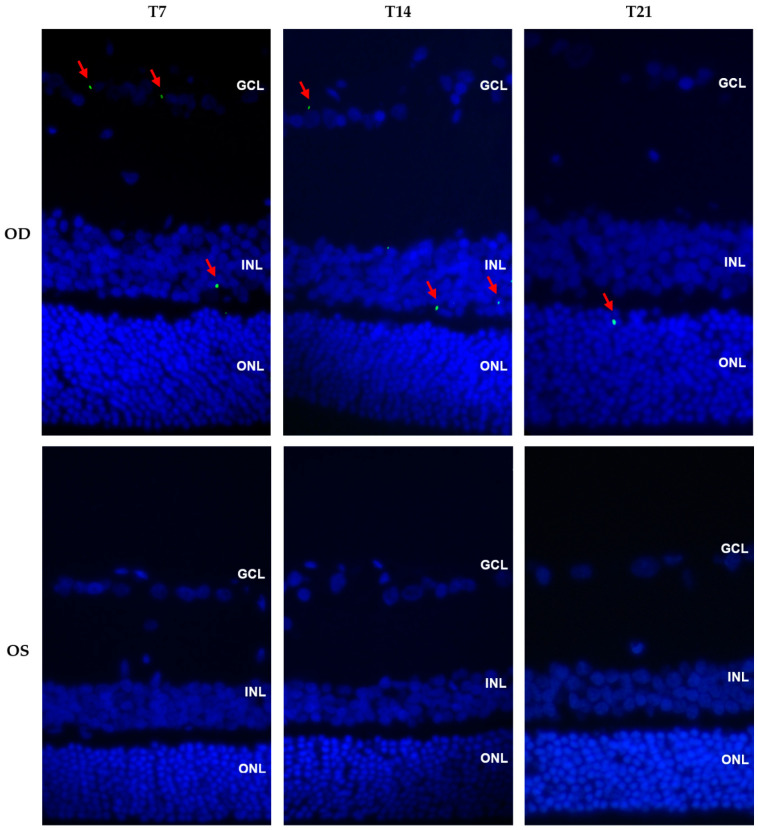
Immunofluorescence photomicrographs showing cross sections of the OD (treated eye) and OS (control eye) in the treatment groups T7, T14, and T21 (magnification 40×). Images show the merging of green and blue filters. Red arrows indicate EPOβ stained in green and cell nuclei are stained in blue with DAPI. GCL, ganglion cell layer; INL, inner nuclear layer; ONL, outer nuclear layer.

Immunofluorescence was performed in cross sections from the OD and OS of the treatment group. EPOβ was detected only in the OD, no EPOβ being observed in the OS of any animal. The number of fluorescent dots (EPOβ) in the ocular tissues was fairly stable throughout the study, although it presented a slight decrease in group T21. EPOβ was detected in the retina from groups T7, T14, and T21, distributed in the GCL, INL, and ONL (Figure 6). EPOβ was also observed in the corneal stroma (T14), corneal endothelium, ciliary body (T21), vitreous, and sclera. The presence of EPOβ in the corneal stroma and in the anterior ocular segment denotes a transcorneal EPOβ permeation. Moreover, the fluorescent signal was still present 21 days after the topical administration of the CS/HA- EPOβ nanoparticles, which suggests a sustained delivery of EPOβ to the eye using this nanoformulation.

## 3. Discussion

Current glaucoma management is based on pharmacological agents and surgical procedures to decrease aqueous humor production or increase its outflow. Yet, research direction is shifting towards neuroprotection as a state-of-the-art glaucoma adjuvant therapy. EPO’s neuroprotective and antiapoptotic effects in ocular tissues were recently described in a comprehensive review article [21], but before that, other studies have also supported additional functions for EPO other than the classical hematopoietic role [22]. Additionally, the retina has been described as an EPO-secreting location with the expression of EPO receptors [23]. Moreover, EPOβ in aqueous solution has already revealed neuroprotective effects in the retina of glaucomatous rats after its subconjuctival administration [9]. 

These findings challenged our team to develop a nanoformulation based on mucoadhesive compounds to facilitate EPOβ transport into the ocular medium after topical instillation. To accomplish this goal, we developed CS/HA-EPOβ nanoparticles, whose *ex vivo* permeation results proved that a larger quantity of EPOβ was retrieved with the nanoparticles [10], compared with the commercial solution (NeoRecormon*^®^,* RocheDiagnostics GmbH, Mannheim, Germany) [24]. Afterwards, we tested the nanoformulation in healthy Wistar Hannover rats using both the subconjunctival [11] and topical (data not published) routes of administration, paving the way for the next logical step of this research: topical administration of CS/HA-EPOβ nanoparticles in a model of retinal disease through a non-invasive, accessible-to-all, and easy-to-administer eyedrop system. In this pre-clinical study in an animal model of glaucoma, we topically administered CS/HA-EPOβ nanoparticles to the rat’s eyes in order to evaluate the permeation of EPOβ in glaucoma conditions, as well as the neuroprotective and neuroregenerative effects of the nanoformulation at the retinal level.

Glaucoma is a painful condition, so the use of analgesia was mandatory to assure animal wellbeing. Meloxicam was selected due to its extended action of pain relieving for 24 h and the absence of contra-indications in the literature that could compromise our results. Pain assessment, according to the Rat Grimace Scale, was classified as not present “0” in all parameters. Animals demonstrated a completely normal behavior throughout the study, suggesting that the analgesic protocol was effective. Furthermore, according to our previous subconjunctival [11] and topical (data not published) studies, the nanocarriers were well tolerated and safe. This led us to believe that the discrete ophthalmological signs observed corresponding to superficial keratitis, a mild degree of conjunctival hyperemia in the operated eyes for 24 to 48 h, and the moderate chromodacryorrhea observed right after the vein coagulation were probably adverse side effects related to the surgery of glaucoma induction and not due to the nanoformulation administration itself. 

At the beginning of the study, the mean IOP value for both groups was 17.3 ± 1.3 mmHg, which is within reference range for healthy Wistars Hannover rats (18.4 ± 0.1 mmHg) [20]. Immediately after glaucoma induction, IOP of the OD increased on average 3.6 folds above physiological values, so cauterization of the three episcleral veins was effective in inducing glaucoma in both the treatment and control groups, as previously described [25,26]. The increase in the IOP values was remarkable in both groups, yet the values returned to normal earlier than expected, which could be due to a sudden increase in the blood outflow in the remaining episcleral vein, promoting the drainage of the aqueous humor. The cauterization of four episcleral veins [26] or other methods described in the literature, such as the circumlimbal suture [27], could be considered in the future to enable a more sustained IOP increase. The IOP of the OS was not influenced by the glaucoma induction in the OD, as mentioned in other studies [25,26,28]. Moreover, the average IOP measurements were similar in both the treatment and control groups, meaning that EPOβ did not interfere with aqueous dynamics or influence IOP values, as already had been reported before [9,29].

ERG traces were an indirect way of proving that glaucoma induction was effective, since they represent the activity of different retinal cells to light stimuli [30], indicating the grade of retinal impairment. The currently used ERG protocol has already been described by others [31,32], and our team has already used it in previous studies [11]. In the dark-adapted ERG in rats, retinal response to dim flashes is mainly composed of negative potentials thought to originate in the inner retina, next to the bipolar cells [33]. This response is called the scotopic threshold response (STR) and is dominated by the retinal ganglion cells (RGCs), which also contribute to the photopic b-wave. RGC damage is directly related to STR and photopic b-wave diminishing [33]. One study reports a STR for rats at ϕ ≈ 5 × 10*^−^*^3^ (−2.30 log cds/m*^−^*^2^) [34], which could be theoretically feasible for us to measure since our SLR begins at −3.02 log cds/m*^−^*^2^. However, the ERG protocol used in this study was not developed to detect the STR; therefore, the photopic b-wave was a more suitable tool to assess the RGC status. Simplifying the ERG interpretation, the b-wave corresponds to the activity of the bipolar and Müller cells, and the a-wave corresponds to the hyperpolarization of cones in the photopic steps and rods in the scotopic steps of the exam [31,35]. The considerably low a- and b-waves recorded from the OD after the microsurgery (day 3) confirmed retinal damage in both groups and, consequently, the success of glaucoma induction. As both waves substantially declined, it indicated that damage occurred in the inner, as well as in the outer, retina of glaucomatous eyes, which is in accordance with the literature [36]. ERG recordings from the OS were kept within normal range after the cauterization, showing no influence of the procedure on the contralateral eye, as previously reported [28]. In addition, both the treatment and control groups presented similar changes in amplitudes and waveforms, denoting the accuracy of the microsurgical procedure. 

To assess the effects of the CS/HA-EPOβ nanoparticles in retinal electric activity, ERG measurements were performed at timepoints 7, 14, and 21 days after glaucoma induction. Overall, electrical retinal response increased with time in both the treatment and control groups. Despite the treatment group presenting higher a-wave and b-wave amplitudes compared to the control group, especially in the scotopic exams, those differences were not statistically significant. However, there were significant differences between day 3 and day 7, 14, and 21, especially in the b-wave from the scotopic exams, precluding that retinal improvement happened earlier and more consistently in the treated animals. In the photopic exams, statistically significant differences were observed only in a few steps of PF, PA, and PLR and, once again, earlier on in the treatment group. Thus, a faster retinal recovery was seen in the treatment group, and the lack of statistical significance might be explained by the reduced sample size at each timepoint (*n* = 4). Moreover, IOP lowering after the cauterization might have contributed to the retinal recovery also observed in the control group. It has been reported by others that in rats, very short-term retinal injury caused by an elevated IOP can be completely reversible within 3 weeks [37]. Taking this into consideration, in future studies, we could modify our glaucoma-inducing technique to better mimic the chronic disease, evaluating CS/HA-EPOβ nanoparticles’ neuroprotective and neuroregenerative effects on a more long-term basis. 

The precocious IOP dropping might also justify the differences found between the photopic and scotopic exams. It is known that the b-wave represents the activity of rod-bipolar cells (RBC) and Müller cells in scotopic conditions [38] and RGC in photopic conditions [33], while the a-wave in photopic conditions indicates the activity of cone-bipolar cells (CBC) [38]. Studies concluded that RBC are less susceptible to acute IOP increases when compared to RGC and CBC. Thus, photopic ERG exams are particularly affected by acute IOP elevations, where CBC and RGC show more cell loss and slower recovery than RBC [33,38,39]. This is consistent with our ERG results, in which the photopic responses showed less improvement than the scotopic ones, this last one probably being related to the RBC recovery. 

Glaucoma causes death of retinal cells, especially of the RGC [40], whose apoptotic process culminates in cell atrophy, chromatin condensation, and nuclear DNA fragmentation [41], potentially leading to a decrease in retinal thickness [37]. In this study, the retinas of the operated eyes presented a generalized thinning compatible with the disease process. Retinas progressively recovered thickness throughout the experiment in both the treatment and control groups, although in the treated rats, this improvement was significantly more marked than in the control group (*p* < 0.05), representing a greater retinal regeneration. This improvement in retinal thickness is in agreement with previous studies that used EPOβ in aqueous solution through subconjunctival administration in glaucomatous rats [9]. Likewise, these findings are consistent with the faster retinal improvement observed in the ERG and apoptosis results of the treated group. EPO is being studied in other models of ocular diseases, such as diabetic retinopathy, retinal vein occlusion, optic neuritis, and ischemic optic neuropathy, also aiming at retinal and optic nerve protection and regeneration [21].

Regarding the immunofluorescence assays, the amount of EPOβ detected in the OD was rather constant through time, and EPOβ was still present in the retina in group T21. In previous topical physiological study, CS/HA-EPOβ nanoparticles were administered during 30 min, 1 drop every 5 min (data not published), while in this topical glaucoma study, nanoparticles were administered during 3 days (1 drop, 3 times a day). Thus, it is understandable that EPOβ permeation followed a more sustained pattern in the present study. In addition, the amount of EPOβ observed in ocular tissues in this study was less than at the timepoints 7, 14, and 21 days in the topical physiological study (data not published). Some factors could contribute to this difference. Not only the topical route of administration led to considerable drug loss [42], but also glaucoma causes the breakdown of blood–ocular barriers (BAB and BRB), which could amplify the clearance of EPOβ, resulting in a lower intraocular EPOβ concentration when compared to non-glaucomatous animals [42,43]. Moreover, the microsurgery for glaucoma induction might have enhanced the natural ocular defense mechanisms, such as reflex blinking and tear production/turnover [2], which would promote a washout effect, decreasing EPOβ permeation. In addition, considering that we proved that EPOβ can reach the retina 12 h after topical administration of CS/HA-EPOβ nanoparticles (data not published), a large amount of EPOβ could be lost in 7 days, which is the first euthanasia timepoint in the glaucoma study. EPOβ was observed in several ocular tissues, including the retinal GCL, INL, and ONL, similar to our physiological study (data not published). This reinforces the present ERG, apoptosis, and retinal measurement results, indicating that the effect of the CS/HA-EPOβ nanoparticles in the retina observed in the treatment group were due to the presence of EPOβ. Likewise, Resende et al. (2018) concluded that EPOβ promoted beneficial effects in the retina of glaucomatous animals after its administration through the subconjunctival route [9]. A transcorneal and sustained permeation of EPOβ through 21 days was observed in the present study, as EPOβ was detected in the corneal stroma in group T14 and in the anterior segment in group T21. This was also found in our physiological study (data not published) and supports the mucoadhesive power of the CS/HA-EPOβ nanoparticles. Lastly, no EPOβ was observed in the OS of any animal, which is in accordance with our previous studies (data not published), meaning that the hypertensive condition did not cause a contralateral absorption of EPOβ.

## 4. Materials and Methods

### 4.1. Materials

Wistar Hannover male rats from Charles River Laboratories (Saint-Germain-Nuelles, France) (n = 24) were used, with an average weight of 329 ± 53 g. Ophthalmological equipment and surgical instruments were available at the Faculty of Veterinary Medicine (ULisboa): Slit Lamp (Hawk Eye^®^, Dioptrix, France), Indirect ophthalmoscope (PanOptic^®^, WelchAllyn, Hillrom, NY, USA), rebound tonometer (Tonolab^®^, Icare, Finland), Electroretinograph (RETIcom, Roland Consult, Stasche & Finger GmbH, Brandenburg, Germany), Surgical microscope (OPMI Lumera i^®^, Carl Zeiss Surgical GmbH, Germany), Phacoemulsification apparatus (Laureate^®^ World Phaco System, Alcon Laboratories, Geneva, Switzerland), and Optical microscope (Olympus^®^ CX 22 RFS1, Olympus, Tokyo, Japan). The epoetin beta used in the nanoformulation was NeoRecormon^®^ 30,000 IU (RocheDiagnostics GmbH, Mannheim, Germany). Hyaluronic acid (eye grade quality; 300 kDa) from Shandong Topscience was a kind gift from Inquiaroma (Barcelona, Spain). Chitosan of low molecular weight (100 kDa, 92% deacetylation, osmolality 290 mOsm/Kg) was acquired from Sigma Aldrich (Irvin, UK). All used drugs were available at the Faculty of Veterinary Medicine (ULisboa), namely, meloxicam (Metacam^®^ 5 mg/mL injectable and Meloxidyl^®^ 0.5 mg/mL oral suspension), ketamine (Ketamidor^®^ 100 mg/mL, Richter Pharma, Wels, Austria), medetomidine (Domtor^®^ 1 mg/mL, Orion Corporation, Espoo, Finland), atipamezole (Antisedan^®^ 5 mg/mL, Zoetis, NJ, USA), sodium pentobarbital (Euthasol^®^ 400 mg/mL, Animalcare Group, North Yorkshire, UK), oxybuprocaine hydrochloride (Anestocil^®^, Edol, Lisbon, Portugal), and carbomer-based gel (Lubrithal^®^, Dechra, Northwich, UK). The 9-0 Vicryl^®^ (Johnson & Johnson^®^, NJ, USA) suture was available at the Faculty of Veterinary Medicine (ULisboa). The slide stainer for hematoxylin and eosin (HE) staining was from Thermo Scientific Gemini™ AS (MA, USA). For immunofluorescence (IF), adhesion slides SuperFrost Plus™ and cover plates (Epredia™, ThermoFisher Scientific, Massachusetts, USA) were used. EPO monoclonal primary antibody 4F11 (MA5-15684) and goat anti-mouse IgG (H + L) secondary antibody DyLight 488 (35502) were from Invitrogen (ThermoFisher Scientific, MA, USA). The blocking reagent (sc-516214) and the aqueous mounting medium with DAPI (sc-2494) were from UltraCruz^®^ (Santa Cruz Biotechnology, TX, USA). The immunofluorescence control used was HepG2 cell cultures (human derived liver hepatocellular carcinoma cell line; ATCC^®^ HB-8065™). Cell culture media and supplements were from Gibco (ThermoFisher Scientific, MA, USA). Histology laboratory devices and reagents were available at the Faculty of Veterinary Medicine (ULisboa). Axioscop 40 fluorescence microscope with an Axiocam HRc camera (Carl Zeiss, Oberkochen, Germany) belonged to the Faculty of Pharmacy (ULisboa).

### 4.2. Methods

Previous to glaucoma induction, all animals (n = 24) underwent a complete ophthalmological examination and an ERG. After glaucoma induction, CS/HA-EPOβ nanoparticles were administered through the topical route, and 3 days later, a control ERG was performed. At selected timepoints, another ERG was made, followed by euthanasia and bilateral enucleation. Histological analysis and apoptosis quantification were the last steps of this study. Procedures are described in detail below.

#### 4.2.1. Animals

Our sample size was calculated by power analysis for unpaired t test, with power of test of 80% and significance level of 0.05, using the software GraphPad StatMate 2 (GraphPad^®^ Software, CA, USA). We chose a sample size of 4 per each timepoint of euthanasia based on a SD of 70 μV for ERG, which was an estimate from the physiological studies [11]. Wistar Hannover male rats (*n* = 24) were randomly split into 6 groups with 4 animals each (*n* = 4), 3 treated groups (T) and 3 control groups (C). According to duration of the experiment, each group was respectively labelled as T7/C7 (one week duration), T14/C14 (two weeks duration), and T21/C21 (three weeks duration). Each group was housed in a conventional EU type IV polycarbonate cage (floor area = 1875 cm^2^) with a stainless-steel wire lid, with food pellets and water ad libitum. Room environment was maintained at 20 ± 2 °C and 50–60% of relative humidity. Luminosity and darkness phases were of 12 h each. This study was performed in accordance with animal ethical requirements, and it was approved by the Organ Responsible for Animal Welfare (Órgão Responsável pelo Bem-Estar dos Animais—ORBEA) of the Faculty of Veterinary Medicine, University of Lisbon, approval date 13 February 2020, code 005/2020, and by the national entity General Directorate of Food and Veterinary (Direção Geral de Alimentação e Veterinária—DGAV), approval date 8 January 2021, code 0421/000/000/2020.

#### 4.2.2. Ophthalmological Examination

At the beginning of the study, all animals (*n* = 24) underwent a complete ophthalmological examination. The neuroophthalmological examination consisted in the evaluation of the palpebral, corneal, pupillary (direct and indirect), and dazzle reflexes, as well as visual acuity through a maze test response. The assessment of the anterior segment was done with a portable Biomicroscope (Hawk Eye^®^, Dioptrix, Toulouse, France), and the posterior segment was assessed with a PanOptic^®^ Ophthalmoscope (WelchAllyn, Hillrom, NY, USA). The IOP measurement was performed with a rebound tonometer (Tonolab^®^, Icare, Finland). These procedures were repeated immediately after glaucoma induction, and then after 1 h, 3 h, and 1, 2, 3, 5, and 7 days in all groups, and at 14 and 21 days in cases where applied.

#### 4.2.3. Electroretinography

In order to evaluate the condition of the retina, animals underwent a flash ERG, which records the activity of rods and cones in response to luminous stimuli. This procedure was performed 3 times in each animal (*n* = 24). The first examination was before glaucoma induction, to confirm normal functioning of the retina. The second exam was 3 days after glaucoma induction, to guarantee that the IOP increase had caused cellular damage to the retinal cells. The third time was at 7 days (Groups T7/C7), 14 days (Groups T14/C14), or 21 days (Groups T21/C21) after glaucoma induction to assess the potential beneficial effects of the CS/HA-EPOβ nanoformulation. After this third ERG, the experiment came to end and euthanasia and enucleation were performed.

**Figure 7:**
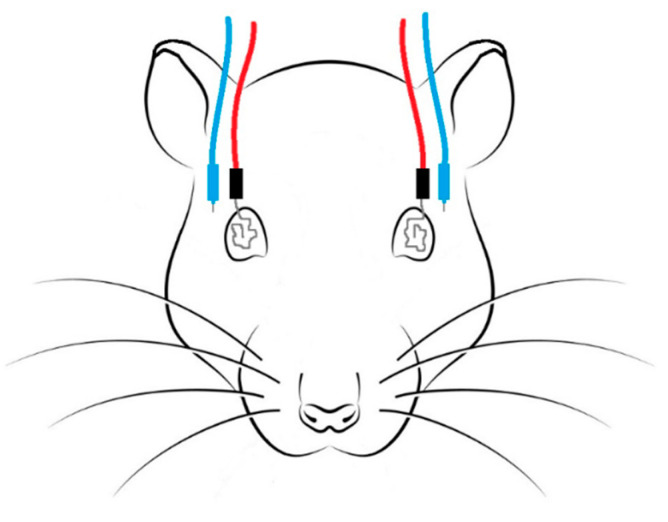
Representation of the ERG setup: active electrodes (red) with silver tips were placed on both corneas, and reference electrodes (blue) were placed subcutaneously between the ears and the lateral canthus ipsilateral to the tested eye.

The ERG protocol was adapted from previously published procedures [31], which demanded a prior scotopic adaptation of 12 h. General anesthesia was mandatory, and a combination of ketamine (70 mg/kg) and medetomidine (0.8 mg/kg) was administered through intraperitoneal injection. To avoid hypothermia, the animal was placed over a heating pad, and its body temperature was periodically measured. One drop of oxybuprocaine hydrochloride (Anestocil^®^, Edol, Lisbon, Portugal) and one drop of a carbomer-based gel (Lubrithal^®^, Dechra, Northwich, UK) was applied onto each cornea. Active silver electrodes were placed in contact with both corneas (Figure 7); reference electrodes were placed between the ear and lateral cantus, subcutaneously (Figure 7); and a ground electrode was placed at the tail base. Retinal responses were recorded simultaneously from both eyes. Light stimulation was achieved by means of a MiniGanzfeld device over the animal’s head, with a base luminescence of 3 cds/m^2^ (0 dB). The reference impedance was <5 kohms, and the light frequency was between 0.1 and 1000 Hz.

The ERG examination was divided into 5 parts, and rod function was tested using dim flashes in scotopic conditions, while cone function was tested using bright flashes and flicker in photopic conditions. In the scotopic luminance response (SLR), light flashes of 9 intensities from −35 dB (–3.02 log cds/m^2^) to +5 dB (0.98 log cds/m^2^) were delivered 3 times per each intensity level, at a frequency of 0.1 Hz. In the photopic adaptation (PA) step, flashes were delivered 3 times after 0, 2, 4, 8, and 16 min of light adaptation, at a frequency of 1.3 Hz, and the intensity was calculated using the maximum b-wave amplitude of the SLR. The photopic luminance response (PLR) used light flashes of 9 intensities, varying from −35 dB to +5 dB, delivered 3 times at a frequency of 1.3 Hz. The photopic flicker (PF) delivered flashes of 0, −5, −10, and −15 dB, at a frequency of 6.3 Hz, after 10 min of light adaptation. Lastly, the scotopic adaptation (SA) used white dim flashes after 0, 2, 4, 8, 16, and 32 min of dark adaptation, delivered 3 times, at a frequency of 1.3 Hz. The entire ERG exam lasted for 75 min, and anesthesia was reverted with an intramuscular injection of atipamezole (2.5 mg/kg).

#### 4.2.4. Preparation of Nanoparticles

Nanoparticles were prepared before glaucoma induction. In a laminar flow cabinet, reagents were sterilized by filtration, using a 0.22 µm filter, and nanoparticles were prepared by a modified ionotropic gelation procedure, based on published protocols [16,17] and previously described by our group [10]. Briefly, to the hyaluronic acid (HA) solution at 1 mg/mL, 1000 IU of EPOβ (NeoRecormon^®^, RocheDiagnostics GmbH, Mannheim, Germany) was added, at room temperature. Then, the HA-EPOβ was added to the chitosan (CS) solution at 1 mg/mL in 0.1% (*v*/*v*) of acetic acid (adjusted pH≈5.5) and NaCl 0.9%, creating CS/HA-EPOβ nanoparticles with a final pH of approximately 6.5. Nanoparticles without EPOβ were called “empty nanoparticles” and were prepared following the same protocol by addition of purified water instead of EPOβ. Nanoparticles’ size, zeta potential (ZP), and polydispersity index (Pdi) were measured afterwards. Empty nanoparticles were administered to the control animals. CS/HA-EPOβ and CS/HA formulations were aspirated with sterilized insulin syringes and kept at room temperature until the topical ocular administration.

#### 4.2.5. Glaucoma Experimental Induction

**Figure 8:**
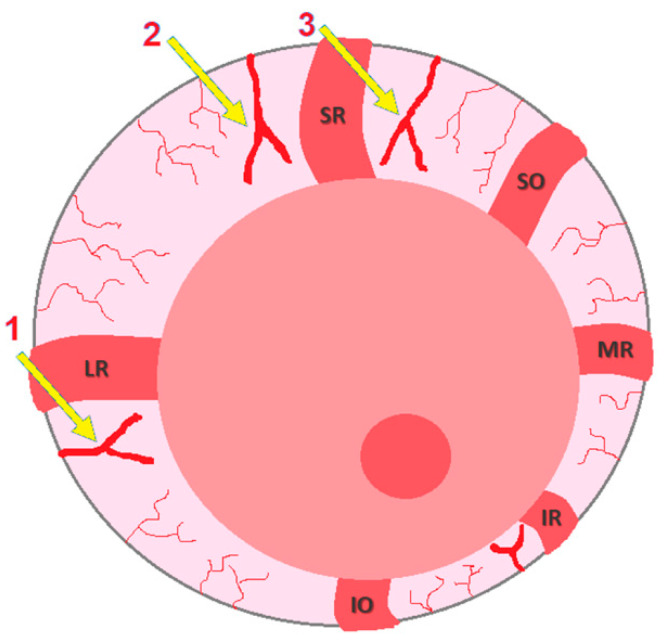
Representation of a rat ocular globe: yellow arrows indicate the cauterized episcleral veins; (1) lateral vein, (2) dorso-lateral vein, (3) dorso-medial vein. Extraocular muscles: SR, superior rectus; SO, superior oblique; MR, medial rectus; IR, inferior rectus; IO, inferior oblique; LR, lateral rectus.

**Figure 9:**
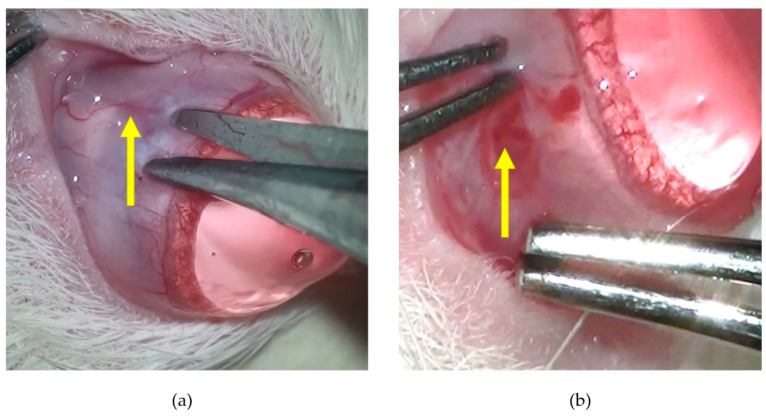
Photo of a rat ocular globe during the microsurgical procedure: (**a**) yellow arrow indicates a dorsal episcleral vein before conjunctiva incision; (**b**) yellow arrow indicates a lateral episcleral vein after the conjunctival incision.

**Figure 10:**
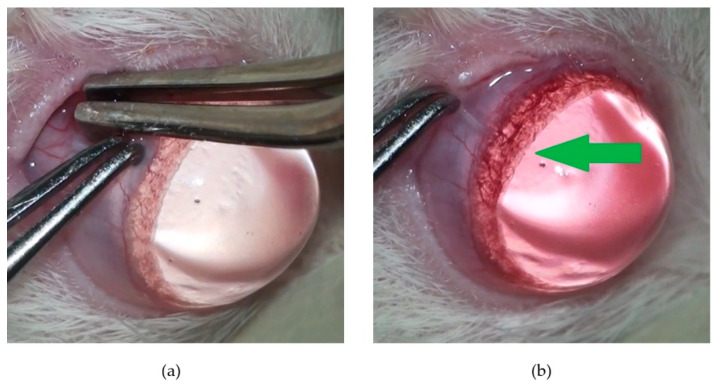
Photo of a rat ocular globe during the microsurgical procedure: (**a**) before the cauterization of the dorsal episcleral vein, showing normal iris vascularization; (**b**) after the cauterization of the dorsal episcleral vein, showing an evident generalized iridal congestion (green arrow).

After the first ERG, animals underwent glaucoma induction in the right eye (OD), while the left eye (OS) was kept intact to remain the control for IOP values. Glaucoma induction was achieved through the cauterization of 3 episcleral veins, whose locations are schematically illustrated in Figure 8. This microsurgical technique was performed using a surgical microscope, under general anesthesia with ketamine (70 mg/kg) and medetomidine (0.8 mg/kg), and using microsurgical instrumentation. Before the surgical procedure, a drop of oxybuprocaine hydrochloride (Anestocil^®^, Edol, Lisbon, Portugal) was applied on the OD, and then a small incision of approximately 2 mm in length was made in the bulbar conjunctiva and Tenon’s capsule, adjacent to the sclero-corneal limbus. Two small radial incisions were made at the edges of the first incision to expose the underlying extraocular muscle, which was gently handled with a clamp to enable the cauterization of the underlying episcleral vein (Figure 9b). Cauterization was performed at the vein bifurcation with the micro-electrocautery attached to the Phacoemulsification apparatus, leading to an immediate iris congestion (Figure 10). This procedure was uniquely performed in the OD, on two dorsal and one lateral veins, and allowed blockage of the respective venous drainage area, leading to an abrupt IOP increase. IOP was measured, on both eyes, immediately before and after the cauterization, to attest if the blockage had been successful. Each conjunctival incision was closed with absorbable 9-0 Vicryl^®^ suture. Continuous hydration of the cornea was performed during the procedure with sterile NaCl 0.9%, and Lubrithal^®^ was applied in the end for corneal lubrication and again 1 h later. For analgesia, meloxicam (1 mg/kg) was administered by subcutaneous route, followed by 1 mg/kg of meloxicam oral suspension on the day after. After the cauterization of 3 episcleral veins, anesthesia was reverted with an intramuscular injection of atipamezole (2.5 mg/kg), and animals were kept in separate cages until full anesthesia recovery. Along with the post-surgical ophthalmological examinations, the presence of discomfort or pain was assessed using the Rat Grimace Scale.

#### 4.2.6. Topical Administration of Nanoparticles

Topical ocular administration of the nanoformulation in the OD was initiated immediately after glaucoma induction with a frequency of one drop 3 times daily during a period of 3 days, to simulate an average frequency of administration of an eyedrop. Groups T7, T14, and T21 received CS/HA-EPOβ nanoparticles transporting EPOβ, while groups C7, C14, and C21 received empty CS/HA nanoparticles. No eyedrop was applied in the contralateral eye (OS). 

#### 4.2.7. Euthanasia and Enucleation

**Figure 11:**
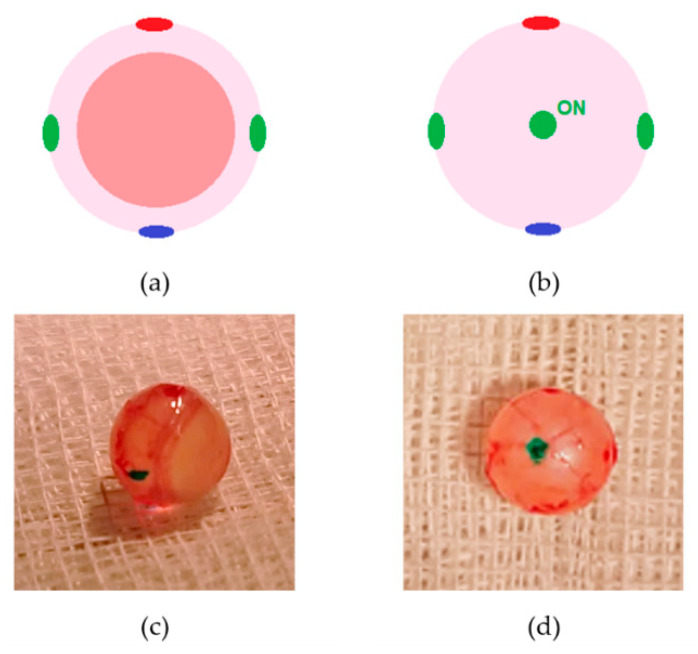
Representation of a rat ocular globe painted with tissue dyes: (**a**) frontal view; (**b**) caudal view. Photographs of a rat ocular globe painted with tissue dyes: (**c**) lateral view; (**d**) caudal view. In green we can identify the transected optic nerve. ON, optic nerve (previously published by our group in Silva et al., 2022 [11]).

On day 7 (T7/C7), day 14 (T14/C14), and day 21 (T21/C21) after topical administration of the nanocarriers, a third ERG was performed, followed by euthanasia with an intraperitoneal injection of sodium pentobarbital (150 mg/kg). Then, both ocular globes were enucleated and painted with tissue dyes at the optic nerve area (green), and also at the lateral (green), medial (green), dorsal (red), and ventral (blue) poles to facilitate eye orientation during paraffin inclusion and histological sections (Figure 11). Ocular globes were fixated in 10% (*v*/*v*) formaldehyde diluted in PBS (0.1 M, pH 7.4), included in paraffin blocks, and processed.

#### 4.2.8. Histological Assessment

After paraffin inclusion, cross sections measuring 3 μm were made with a microtome, in a total of 4 sections for immunofluorescence and 4 sections for hematoxylin and eosin (HE) staining. Slides were kept at 60 °C for 1 h and then overnight at 37 °C. HE slides were processed in the multi-tasking stainer Gemini™ AS, and then observed in an optical microscope (Olympus^®^ CX 22 RFS1, Olympus, Tokyo, Japan) in order to evaluate cellular structure and perform measurements of the retinal thickness from a distance of 500 µm until 1500 µm away from the optic nerve, which corresponds approximately to two microscopic view fields. Pictures were taken from each field at 40× magnification and, posteriorly, 10 measurements of the retinal thickness from the inner limiting membrane (ILM) to the outer limiting membrane (OLM) were performed in each picture using the ImageJ^®^ Software. 

Immunofluorescence slides followed a more complex protocol, starting from being deparaffinized in xylol, and gradually rehydrated in alcohol from 100° to 70°, and finally in purified water. Sections were washed with Triton X-100 solution (0.1% *v*/*v* in PBS) and Tween 20 solution (0.1% *v*/*v* in PBS), followed by setting the slides in cover plates. Meanwhile, HepG2 cells (positive control for immunofluorescence) were submitted to a hypoxic environment during 2 h at 37 °C, then fixated in 10% (*v*/*v*) formaldehyde in PBS and washed with Triton X-100 solution. From that point on, HepG2 cells followed the same protocol as the cross sections. Sections were incubated with UltraCruz^®^ Blocking Reagent (Santa Cruz Biotechnology, TX, USA) for 1 h at room temperature, followed by a washing step and incubation with EPO monoclonal primary antibody 4F11 (1:400) overnight at 4 °C. Then, another washing step was made, followed by incubation with goat anti-mouse secondary antibody DyLight 488 (Invitrogen, ThermoFisher Scientific, MA, USA) (1:1000) in the dark for 1 h, at room temperature. Slides were carefully disassembled from the cover plates and received a small amount of UltraCruz^®^ mounting medium with DAPI, followed by a coverslip and varnish sealing. The immunofluorescence analysis was qualitative and blindly performed by the same investigator using an Axioscop 40 fluorescence microscope with an Axiocam HRc camera (Carl Zeiss, Oberkochen, Germany ), and images were processed with the AxioVision software (Rel.4.8.1, Carl Zeiss, Oberkochen, Germany). EPOβ fluorescence was detected in green, the negative control being the OS and the positive control for the immunofluorescence technique being the HepG2 cells.

#### 4.2.9. Statistical Methods

Statistical analysis was performed with GraphPad Prism version 6.0 (GraphPad Software, CA, USA) and Microsoft Office Excel (Microsoft, WA, USA), using one-way ANOVA and paired *t*-test to detect significant differences between group means. The statistical significance was 95%, corresponding to a *p*-value of 0.05. Results were presented as mean ± standard deviation (SD).

## 5. Conclusions

Beforehand, our team developed CS/HA-EPOβ nanoparticles and administered them through both subconjunctival and topical ocular routes in healthy rats, confirming EPOβ retinal delivery in physiological conditions using this nanoformulation. Being so, we considered it opportune to explore the topical route of CS/HA-EPOβ nanoparticles administration in glaucomatous rats and to assess their safety and potential neuroprotective effects. The present study revealed that ocular topical administration of this newly developed nanoformulation allowed for EPOβ permeation, inclusive through the transcorneal pathway, and ultimately reaching the inner retina. Improvements in retinal electrical activity and in retinal thickness were observed earlier and more expressively in the treated animals. Not only did we prove that EPOβ could be delivered to the retina, in glaucomatous rats, by topical administration of these new CS/HA-EPOβ nanoparticles, but we have also demonstrated that EPOβ elicited retinal neuroprotective effects in these experimental conditions. 

No other studies on topical ocular administration of nanocarriers with neuroprotective drugs in a glaucoma animal model have been reported so far in the literature. Although adjustments in the experimental design should be addressed to lead to more statistically significant differences between the treated and control groups, the results of this preclinical study are promising and contribute to innovative insights into the field of ocular drug delivery. Our results open the window to the possibility of delivering neuroprotective compounds by the topical route of administration, targeting the posterior segment of the eye, which could become a user-friendly, cheap, safe, and effective way to help preserve visual acuity in ischemic or degenerative retinopathies, namely, in glaucoma, both in humans and in animals.

## Figures and Tables

**Table 1:** IOP measurements (mmHg) for both eyes, before and after (T = 0) glaucoma induction, in treatment (*n* = 12) and control groups (*n* = 12). OD, right eye; OS, left eye.

	Treatment (mmHg)		Control (mmHg)	
	Before	After (T = 0)	Before	After (T = 0)
**OD**	10.9 ± 0.8 *	62.6 ± 8.3 *	11.1 ± 0.8 *	63.6 ± 7.9 *
**OS**	10.8 ± 0.7	10.7 ± 7.9 *	10.8 ± 0.7	11.0 ± 1.2 *

* *p* < 0.05.

**Table 2:** ERG results from the OD: a-waves and b-waves recorded during the five ERG components. Results are presented as mean ± SD in microvolts (μV). ERGs were performed in the treatment and control groups, before glaucoma induction and again 3 days later.

		Treatment (μV)				Control (μV)			
		Before		3 days		Before		3 days	
		a-wave	b-wave	a-wave	b-wave	a-wave	b-wave	a-wave	b-wave
**SLR**	−35 (dB)	18 ± 4 *	293 ± 110 *	5 ± 4 *	41 ± 24 *	18 ± 4 *	296 ± 76 *	7 ± 3 *	43 ± 28 *
	−30	22 ± 10 *	324 ± 107 *	10 ± 5 *	78 ± 41 *	21 ± 10 *	326 ± 85 *	10 ± 6 *	72 ± 31 *
	−25	26 ± 11 *	336 ± 122 *	11 ± 6 *	80 ± 46 *	28 ± 8 *	341 ± 91 *	12 ± 8 *	78 ± 51 *
	−20	41 ± 13 *	380 ± 119 *	14 ± 7 *	86 ± 51 *	37 ± 15 *	391 ± 78 *	14 ± 8 *	86 ± 28 *
	−15	76 ± 31 *	419 ± 140 *	17 ± 20 *	90 ± 53 *	79 ± 18 *	428 ± 82 *	17 ± 13 *	89 ± 54 *
	−10	152 ± 69 *	488 ± 126 *	28 ± 22 *	93 ± 55 *	156 ± 39 *	496 ± 80 *	29 ± 17 *	91 ± 59 *
	−5	204 ± 51 *	578 ± 144 *	33 ± 17 *	96 ± 69 *	210 ± 24 *	579 ± 99 *	34 ± 14 *	97 ± 68 *
	0	231 ± 68 *	602 ±159 *	48 ± 20 *	124 ± 73 *	240 ± 31 *	612 ± 114 *	43 ± 15 *	121 ± 46 *
	5	263 ± 36 *	618 ± 145 *	46 ± 27 *	124 ± 67 *	266 ± 31 *	627 ± 119 *	45 ± 15 *	123 ± 47 *
**PA**	0 (min)	18 ± 9	202 ± 48 *	12 ± 8	71 ± 29 *	17 ± 13	206 ± 60 *	10 ± 9	68 ± 28 *
	2	23 ± 20	211 ± 37 *	16 ± 10	75 ± 41 *	22 ± 15	198 ± 36 *	15 ± 9	77 ± 40 *
	4	18 ± 13	213 ± 41 *	13 ± 6	79 ± 56 *	21 ± 13	199 ± 42 *	14 ± 10	75 ± 23 *
	8	22 ± 15	212 ± 49 *	14 ± 16	71 ± 38 *	22 ± 15	200 ± 42 *	14 ± 13	73 ± 26 *
	16	28 ± 16	205 ± 53 *	16 ± 13	64 ± 24 *	27 ± 17	199 ± 49 *	16 ± 9	67 ± 23 *
**PLR**	−35 (dB)	19 ± 12	70 ± 24 *	13 ± 12	41 ± 21 *	17 ± 9 *	71 ± 25 *	11 ± 9 *	42 ± 22 *
	−30	25 ± 11 *	73 ± 36 *	12 ± 11 *	45 ± 19 *	25 ± 13 *	79 ± 42 *	12 ± 13 *	47 ± 23 *
	−25	27 ± 14	82 ± 30 *	20 ± 15	49 ± 23 *	27 ± 15 *	85 ± 39 *	11 ± 7 *	49 ± 19 *
	−20	25 ± 13	84 ± 29 *	15 ± 13	49 ± 22 *	26 ± 14 *	89 ± 35 *	14 ± 4 *	52 ± 20 *
	−15	22 ± 15	94 ± 32 *	17 ± 12	51 ± 18 *	21 ± 14	89 ± 37 *	21 ± 8	53 ± 14 *
	−10	17 ± 13	107 ± 36 *	17 ± 11	56 ± 28 *	21 ± 18	110 ± 32 *	14 ± 15	55 ± 16 *
	−5	27 ± 25	127 ± 40 *	13 ± 11	58 ± 23 *	24 ± 23	128 ± 50 *	20 ± 14	61 ± 21 *
	0	24 ± 22	198 ± 64 *	12 ± 9	59 ± 24 *	27 ± 22	202 ± 80 *	13 ± 9	60 ± 23 *
	5	23 ± 30	227 ± 53 *	10 ± 11	71 ± 19 *	22 ± 28	218 ± 96 *	12 ± 15	73 ± 21 *
**PF**	0 (dB)	7 ± 4	229 ± 47 *	5 ± 4	51 ± 23 *	8 ± 6	239 ± 59 *	6 ± 4	52 ± 10 *
	−5	13 ± 9	134 ± 33 *	9 ± 5	47 ± 21 *	18 ± 8 *	122 ± 41 *	11 ± 6 *	47 ± 22 *
	−10	19 ± 13	81 ± 32 *	13 ± 8	44 ± 13 *	20 ± 17	85 ± 30 *	13 ± 8	43 ± 16 *
	−15	18 ± 9	61 ± 24 *	16 ± 8	40 ± 12 *	23 ± 15	71 ± 24 *	14 ± 9	39 ± 15 *
**SA**	0 (min)	25 ± 14 *	243 ± 52 *	13 ± 8 *	55 ± 23 *	25 ± 13 *	241 ± 70 *	12 ± 9 *	53 ± 28 *
	2	42 ± 24 *	265 ± 53 *	16 ± 8 *	57 ± 30 *	41 ± 20 *	266 ± 44 *	17 ± 8 *	60 ± 34 *
	4	47 ± 30 *	291 ± 57 *	18 ± 13 *	67 ± 30 *	46 ± 23 *	285 ± 72 *	18 ± 10 *	62 ± 35 *
	8	61 ± 43 *	333 ± 67 *	19 ± 9 *	69 ± 36 *	57 ± 32 *	329 ± 80 *	19 ± 11 *	72 ± 40 *
	16	71 ± 48 *	365 ± 41 *	28 ± 16 *	79 ± 31 *	80 ± 46 *	355 ± 76 *	26 ± 13 *	77 ± 51 *
	32	91 ± 49 *	403 ± 52 *	39 ± 26 *	82 ± 59 *	85 ± 56 *	405 ± 123 *	39 ± 26 *	83 ± 38 *

* *p* < 0.05. SLR, scotopic luminescence response; PA, photopic adaptation; PLR, photopic luminescence response; PF, photopic flicker; SA, scotopic adaptation; dB, decibel; min, minutes.

**Table 3:** ERG results of the OD after glaucoma induction: b-wave mean amplitudes recorded in a single step of each ERG part, presented as mean ± SD in microvolts (μV). ERGs performed in the treatment (T) and control (C) groups 3, 7, 14, and 21 days after glaucoma induction.

	b-Wave (μV)							
	3 days		7 days		14 days		21 days	
	T	C	T	C	T	C	T	C
**SLR** **(5 dB)**	124 ± 66	123 ± 47	216 ± 90	212 ± 97	247 ± 91	233 ± 80	341 ± 55	244 ± 80
**PA** **(16 min)**	64 ± 24	67 ± 23	95 ± 25	82 ± 18	104 ± 24	102 ± 46	125 ± 50	115 ± 68
**PLR** **(5 dB)**	71 ± 19	73 ± 21	93 ± 48	84 ± 23	118 ± 59	107 ± 35	129 ± 42	118 ± 32
**PF** **(0 dB)**	51 ± 23	52 ± 17	76 ± 51	58 ± 33	89 ± 19	82 ± 43	97 ± 52	94 ± 29
**SA** **(32 min)**	82 ± 59	83 ± 38	125 ± 45	103 ± 40	162 ± 32	135 ± 44	176 ± 65	139 ± 41

SLR, scotopic luminescence response; PA, photopic adaptation; PLR, photopic luminescence response; PF, photopic flicker; SA, scotopic adaptation; dB, decibel; min, minutes.

**Table 4:** ERG (b-wave) comparison between timepoints T3 and T7, T14 and T21 after glaucoma induction in the OD, both in treated and control groups. Results show the ERG steps (in dB or min) with statistically significant differences (*p* < 0.05) between timepoints.

	***b-Wave (μV)***					
	3–7 days		3–14 days		3–21 days	
	T	C	T	C	T	C
*SLR* (dB)	−25 −20 −15 −10 −5 0 5	−5 0	−25 −20 −15 −10 −5 0 5	−10 −5 0	−35 −30 −25 −20 −15 −10 −5 0 5	−25 −20 −15 −10 −5
*PA* (min)	ns	ns	ns	ns	8 16	ns
*PLR* (dB)	ns	ns	ns	ns	0 5	ns
*PF* (dB)	ns	ns	0	0	0 −5 −10	0
*SA* (min)	0 2 4 8 16	0 2 4	0 2 4 8 16 32	0 2 4 8 16	0 2 4 8 16 32	0 2 4 8 16 32

ns, *p* > 0.05; T, treated groups; C, control groups; SLR, scotopic luminescence response; PA, photopic adaptation; PLR, photopic luminescence response; PF, photopic flicker; SA, scotopic adaptation; μV, microvolts; dB, decibel; min, minutes.

**Table 5:** Retinal thickness measurements (µm) of the OD from treatment (*n* = 12) and control groups at 7, 14, and 21 days (*n* = 12) measured in a distance between 500 µm and 1500 µm from the optic nerve.

	Treatment (µm)	Control (µm)
**7 days**	104.3 ± 21.1 *	92.3 ± 13.3 *
**14 days**	122.2 ± 13.2 *	110.8 ± 7.0 *
**21 days**	145.6 ± 22 *	120.2 ± 10.6 *

* p < 0.05.

## Data Availability

Data is contained within the article.
